# Sedation at Sea of Entangled North Atlantic Right Whales (*Eubalaena glacialis*) to Enhance Disentanglement

**DOI:** 10.1371/journal.pone.0009597

**Published:** 2010-03-09

**Authors:** Michael Moore, Michael Walsh, James Bailey, David Brunson, Frances Gulland, Scott Landry, David Mattila, Charles Mayo, Christopher Slay, Jamison Smith, Teresa Rowles

**Affiliations:** 1 Biology Department, Woods Hole Oceanographic Institution, Woods Hole, Massachusetts, United States of America; 2 Department of Large Animal Clinical Sciences, College of Veterinary Medicine, University of Florida, Gainesville, Florida, United States of America; 3 Veterinary Specialty Team - Sedation, Pain Management and Anesthesiology, Pfizer Inc., Madison, Wisconsin, United States of America; 4 Veterinary Science, The Marine Mammal Center, Sausalito, California, United States of America; 5 Marine Animal Entanglement Response, Provincetown Center for Coastal Studies, Provincetown, Massachusetts, United States of America; 6 Hawaiian Islands Humpback Whale National Marine Sanctuary, National Ocean Service, National Oceanographic Atmospheric Administration, Kihei, Hawaii, United States of America; 7 Coastwise Consulting Inc., Athens, Georgia, United States of America; 8 Protected Resources Division, National Marine Fisheries Service, National Oceanographic Atmospheric Administration, Gloucester, Massachusetts, United States of America; 9 Marine Mammal Conservation Division, National Marine Fisheries Service, National Oceanographic Atmospheric Administration, Silver Spring, Maryland, United States of America; Institut Pluridisciplinaire Hubert Curien, France

## Abstract

**Background:**

The objective of this study was to enhance removal of fishing gear from right whales (*Eubalaena glacialis*) at sea that evade disentanglement boat approaches. Titrated intra muscular injections to achieve sedation were undertaken on two free swimming right whales.

**Methodology/Principal Findings:**

Following initial trials with beached whales, a sedation protocol was developed for right whales. Mass was estimated from sighting and necropsy data from comparable right whales. Midazolam (0.01 to 0.025 mg/kg) was first given alone or with meperidine (0.17 to 0.25 mg/kg) either once or four times over two hours to whale #1102 by cantilevered pole syringe. In the last attempt on whale #1102 there appeared to be a mild effect in 20–30 minutes, with duration of less than 2 hours that included exhalation before the blowhole fully cleared the water. Boat avoidance, used as a measure of sedation depth, was not reduced. A second severely entangled animal in 2009, whale #3311, received midazolam (0.03 mg/kg) followed by butorphanol (0.03 mg/kg) an hour later, delivered ballistically. Two months later it was then given midazolam (0.07 mg/kg) and butorphanol (0.07 mg/kg) simultaneously. The next day both drugs at 0.1 mg/kg were given as a mixture in two darts 10 minutes apart. The first attempt on whale #3311 showed increased swimming speed and boat avoidance was observed after a further 20 minutes. The second attempt on whale #3311 showed respiration increasing mildly in frequency and decreasing in strength. The third attempt on whale #3311 gave a statistically significant increase in respiratory frequency an hour after injection, with increased swimming speed and marked reduction of boat evasion that enabled decisive cuts to entangling gear.

**Conclusions/Significance:**

We conclude that butorphanol and midazolam delivered ballistically in appropriate dosages and combinations may have merit in future refractory free swimming entangled right whale cases until other entanglement solutions are developed.

## Introduction

Human impacts on North Atlantic right whales (*Eubalaena glacialis*) have largely erased potential population growth despite robust recent calving rates [1]. The major traumas found at necropsy have been propeller cuts and blunt trauma from vessels, and constrictive trauma from fishing gear entanglement [2]. Since first pioneered by Jon Lien in Canada in the 1970's [3], whale disentanglement techniques have been attempted mostly on animals anchored by fishing gear. Newer techniques developed in the 1980's by the Provincetown Center for Coastal Studies, Provincetown, MA, USA, (PCCS) are now being used to disentangle free-swimming entangled whales. These techniques rely primarily on physical restraint by adding buoyancy and drag to the trailing gear. Using small boats, buoys and drogues in an effort to tire and restrict movement of the whale, entangling gear can be removed with relative safety using pole-mounted knives. Disentanglement success rates have varied by species and entanglement configuration, with some species being more tractable than others: critically endangered right whales being the least tractable. Free-swimming right whale entanglement cases exhibiting wraps of line far forward on the body, in areas such as the rostrum and flippers, have the lowest disentanglement success rate to date (PCCS unpublished data). The primary position for attempting to cut line in a free swimming whale has been from behind the tail within a boat towed by the whale, as this is a position of relatively higher human safety. However this position often greatly limits access to entangling lines. In 1999 an entangled right whale catalogued by the New England Aquarium as #2030 (http://rwcatalog.neaq.org) proved to be refractory to multiple disentanglement attempts. It was first sighted entangled on May 10 1999 with fishing gear stretched taught between both axillae that had stripped off large pieces of dorsal blubber and skin. The disentanglement team requested assistance to deliver antibiotics to the whale once it had been disentangled. A pole syringe based on a cantilevered pole system [4] was prepared and attempts to physically restrain the animal using a tail harness were made by PCCS and others. The animal was never disentangled, and drugs were not delivered. The whale eventually died 5 months later. Animal welfare and conservation concerns in this case initiated the question of planning for medical intervention in similar cases. The possibility of sedating animals to enhance disentanglement was discussed. There is a history of the use of sedatives in captive marine mammals [5] where benzodiazepines or meperidine have enabled stressful procedures such as gastroscopy and bronchoscopy [6], [7]. Despite earlier predictions of the dire consequences of using immobilizing drugs in free swimming animals [8], an initial attempt to deliver sedatives to a right whale has been briefly reported [9]. Here we describe the details of that attempt, as well as a subsequent right whale sedation and disentanglement.

## Methods

### Delivery Systems

**Figure 1:**
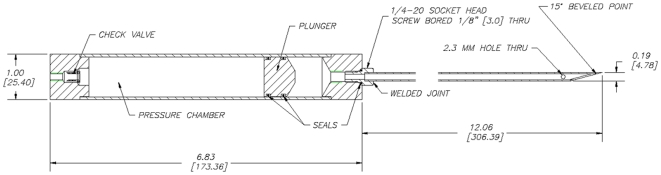
Schematic of polycarbonate pole syringe for injecting a large whale at sea (dimensions in inches (mm). The Paxarms ballistic syringe operates on a similar principle.

A pole syringe was developed for use on right whale #1102 because existing ballistic drug delivery systems for terrestrial species had inadequate volume for such large animals. A cantilevered pole system, originally designed for measuring blubber thickness acoustically [4] was adapted to be practical in a small inflatable boat, with an open pivot to allow immediate release from the boat if need be. A polycarbonate syringe was fabricated with a check valve, plunger and a stainless steel needle (Figure 1). The pressure chamber was filled with butane to a pressure at which the butane condensed to a liquid. The side injection port on the distal portion of the needle carried a tygon tube sleeve that was displaced upon entry into the animal allowing the butane under pressure to advance the plunger and inject the drug. A carcass trial was conducted to establish the weight and elevation of needle holder required to ensure efficient muscle penetration.

Simpler systems, such as a hand held pole, a crossbow, and a spear gun were tested and rejected on grounds of safety.

A ballistic syringe (Paxarms, 37 Kowhai St, Timaru, New Zealand), was developed for use on right whale #3311. The dart portion of this system operates on the same principle as the syringe illustrated in Figure 1. The syringe body comprised a 33 cm long by 19 mm diameter anodized aluminum tube threaded at both ends, machined to have an O ring seat. The flight end cap has a valve to allow pressurization of air behind the plunger. The 30 cm long×6.4 mm outside diameter stainless steel needle is lined with a carbon fiber tube and has 3 end ports. A rubber stop screws over the end piece. All O rings as well as the syringe and projector barrel are coated with silicon lubricant. The projector is a modified.22 caliber rifle, firing blanks custom filled by Paxarms. An adjustment of a valve controlling air flow in to the expansion chamber allows for compensation of the firing power dependent on range of firing. This adjustment is linked to the sighting scope such that the sight compensates for the required trajectory. A stainless steel leader is attached via a swivel to the rear of the syringe. The leader runs up corresponding grooves in the syringe and projector barrel to be tied to 20 m of 80 kg test line wound around the projector barrel tip, and then tied to a 22.86 cm (9in) long by 7.62 cm (3in) wide cm styrofoam ‘Shark’ float. The line windings on the barrel are lightly wrapped with office sticky tape to allow it to peel off once the dart is fired. The dart is fired at a range of about 15 m. The line spins off the barrel and the float is left to trail behind the whale until the drag on the float extracts the needle.

### Sedatives

Midazolam (a benzodiazepine anxiolytic, amnestic, hypnotic, anticonvulsant and muscle relaxant) in ethanol was compounded for the initial trials on whale #1102 by University of Wisconsin Veterinary School Pharmacy (2015 Linden Drive, Madison, WI, USA). Use of the commercial drug at 5 mg/ml was impractical, so the midazolam base was concentrated in ethanol to reduce the total volume administered. Meperidine (an opioid analgesic) was supplied by University of Wisconsin Veterinary School Pharmacy. The normal commercial grade drug is available at 100 mg/ml. For this attempt it was concentrated to 550 mg/ml. An aqueous solution of midazolam HCl was also used, compounded by ZooPharm (Box 2023, Fort Collins, CO, USA). Butorphanol (an opioid analgesic) was supplied by ZooPharm and could be mixed with midazolam HCl. Concentrations used are given in Table 1.

**Table 1:** Summary of Sedation Dosing of Two Right Whales.

Date	Time	Drug	Concen-tration (mg/ml)	Assumed weight (kg)	Dose mg/kg	Result
Right Whale # 1102 (Year 2001)						
Jun 26^th^		Midazolam	90	45000	0.01	No effect
Jul 14th		Midazolam	90	45000	0.025	No effect
		Meperidine	550		0.017	
Aug 30^th^	Over 2 h.	Midazolam	90	40000	0.1	Less forceful surfacings, and exhaling before the blowhole was clear of the water with a less powerful blow.
		Meperidine	550		1	
Right Whale # 3311 (Year 2009)						
Jan 23^rd^	1433	Midazolam	70	20000	0.028	Increased speed and avoidance 20 minutes later
	1533	Butorphanol	50		0.033	
Mar 5^th^	1418	Midazolam	50	20000	0.071	Oblique?did not penetrate subdermal sheath. Respiration increased frequency, decreased depth. Not statistically signficant. No reduction in boat aversion
	1418	Butorphanol	50		0.071	
Mar 6^th^	1133 to 1143	Midazolam	50	20000	0.1	Marked reduction of boat avoidance. Respiration increased frequency, decreased depth.
	1143	Butorphanol	50		0.1	

### Reversal

Naltrexone (ZooPharm 50 mg/ml) has been used to reverse the sedative effects of butorphanol in other cetaceans [10], [11] at 0.005 mg/kg to 0.3 mg/kg i/m (intra-muscular) and was available in the event of problems with the sedation.

### Ethics

**Figure 2:**
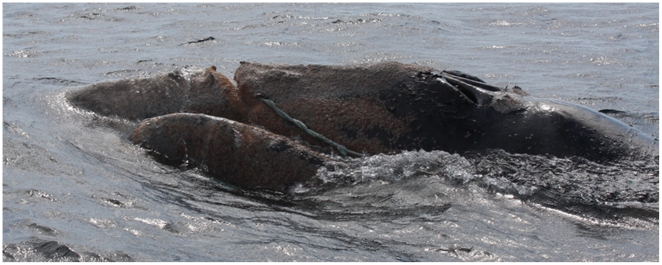
Image of rope cutting in to the head of right whale #3311. This whale trailed line that cut into the upper jaw. The curved organ below the line is the left lip. The double slotted blowhole is evident at right. The brown color covering the skin is a mass of cyamid whale lice typical of a right whale in very poor condition. Photograph credit – Florida Wildlife Research Institute.

This research was undertaken after approval and permitting by the National Oceanic Atmospheric Administration Office of Protected Resources under the Marine Mammal Protection Act and Endangered Species Act. Enhancing the welfare of the subject, severely entangled animals, was the primary agenda of the research and in the case of whale #3311 (Figure 2), welfare was perhaps significantly improved by the procedure described.

### Strategy

Given the many unknowns in terms of doses and effects it was agreed that the only animals that would be considered for sedation were those that would likely die, if not disentangled, and could not be not disentangled by more traditional means; and that could be telemetry-tracked prior to sedation attempts, for visual health assessments and planning. The vessel used for sedation of whale #3311 was a 7.2 m rigid hull inflatable boat (RHIB) operating one of two 90 hp four cycle outboard motors, with the projection system being operated from a platform 2.5 m above sea level. The disentanglement vessel approaching whale #3311 was a 5.5 m RHIB with a 90 hp 4 cycle outboard motor. Whale weights were estimated on the basis of available necropsy and catalog data [2].

### Experimental Design

Delivery system and drug trials were undertaken sequentially on whales that were beached, trapped in a river and at sea. Drug use and delivery protocols were iteratively developed through a series of 5 consecutive trials, evolving the treatment regimen in light of accrued findings.

## Results

### Trial 1

An emaciated juvenile gray whale (*Eschrichtius robustus*) beached in ventral recumbency along the California coast on April 8^th^ 2000. It was824 cm in length, 264 cm in axillary girth,with a ventral axilla blubber depth of 75 mm, and with an intact blink reflex. It was hand injected with 25 cc of 51 mg/ml midazolam i/m into the lateral periaxial lumbar area mid body using a 7.6 cm 18 G spinal needle. Twelve minutes later, the blink reflex was diminished, 29 minutes after injection the peduncle could be lifted with no response and a blood sample taken. The animal was euthanased 38 minutes after injection. The estimated weight of this animal was of the order of 5000 kg [12]. Thus the dose given was of the order of 0.025 mg/kg.

### Trial 2

Right whale #1102 was observed by an aerial survey on June 8, 2001 to have a severe head entanglement, with associated deep incision across the rostrum. A telemetry buoy was attached to the trailing gear by PCCS on June 9th. The animal proceeded to move extensively in the Gulfs of Maine and St Lawrence. The first major disentanglement effort was in the Great South Channel southeast of Nantucket, MA, USA, June 26th, 2001, at which time the condition of the animal had significantly deteriorated. The whale was assumed to weigh 45,000 kg. Midazolam and meperidine were chosen for these initial attempts based on successful use of midazolam alone or in combination with meperidine in other cetaceans (Walsh, personal communication, clinical records and [6]). Balancing the potential difference in metabolism from previous use in other species it was decided to start with lower relative dosages and one drug. The dose of midazolam administered was 0.01 mg/kg and again 37 minutes later using the cantilevered pole syringe [4]. There was no evident effect on the animal. Meperidine (an opioid analgesic) was then added at the second disentanglement attempt on July 14^th^. Midazolam was given at 0.025 mg/kg. The meperidine dose given was 0.17 mg/kg. There was no evident effect on the animal. On August 30^th^ a third attempt was made assuming a 40,0001kg whale using a) midazolam at 0.025 mg/kg, at a concentration of 90 mg/ml, giving an 11 ml/dose and b) meperidine at 0.251mg/kg, at a concentration of 550 mg/ml giving an 18 ml dose. The above combined dose was given four times in 2 hours. There appeared to be an onset of effect in 20–30 minutes with duration of effect of less than 2 hours. The level of central nervous system depression appeared to be low. The animal did however appear to some, but not others, to be mildly sedated, exhibiting less forceful surfacings, and exhaling before the blowhole was clear of the water with a less powerful blow. The animal was however not disentangled on any of these attempts. Dosing information for this and a subsequent case are given in Table 1. The last satellite transmission was on Sept. 16^th^ 2001, presumably when the whale and telemetry buoy sank.

### Trial 3

After land and carcass based trials demonstrated the apparent utility of the Paxarms system, it was used to successfully deliver antibiotics to two free swimming humpback whales (*Megaptera novaeangliae*) in the Sacramento River, CA USA on May 26^th^ 2007 [13]. It deployed well, and the darts detached from the animals within 2 to 24 hours.

### Trial 4

The Paxarms system was also used to administer 50 ml of 600 mg/ml meperidine as a pre euthanasia drug to a moribund beached sperm whale (*Physeter macrocephalus*) at Terra Ceia, Manatee, FL, USA on January 1^st^ 2008. The system performed well though the level of sedation achieved was not easy to ascertain as a result of the depressed condition of the animal.

### Trial 5

The Paxarms system was first deployed for a series of sedation attempts on a chronically entangled right whale #3311, an animal of unknown sex, born in 2003 (Figure 2). After being observed gear free on April 21^st^ 2008, the animal was first reported entangled on January 14^th^ 2009 18 miles off Brunswick GA, USA. After being assessed with a fatal entanglement and serious injuries at the rostrum and lower lip, a telemetry tag was attached to the trailing entangling gear that afternoon. Body mass was estimated at 20,000 kg from a published age/mass curve [2]. On January 23^rd^ 2009, 15 nm east of St Augustine Inlet midazolam in ethanol was given at 0.028 mg/kg and butorphanol hydrochloric acid (HCl) at 0.033 mg/kg (Table 1). Increased speed and avoidance was noted 20 minutes later. Conditions precluded a further dosing attempt. The two drugs were immiscible given their ethanol vs. water base, thus this combination was less desirable for future administration where volume would be a limiting factor.

March 5^th^ 2009 – Right whale #3311 was sighted 15.6 nm NE of Ponce de Leon Inlet, FL, USA. Respirations were recorded starting at 14:09. A dart loaded with 0.071 mg/kg midazolam HCl and butorphanol HCl was delivered at 14∶18. Respirations per five minutes increased from 3 to 5 before darting to 5 to 7 after darting – a notable but statistically insignificant change. At the same time respiration depth appeared to decrease. At times exhalation began before the blowhole was clear of the water. There was no reduction in the evasiveness of the animal to boat approaches and no cuts to disentangle could be made. The angle of insertion of the dart was shallow. Thus it was unclear if all or some of the drug was injected into the blubber rather than the muscle, potentially delaying and or reducing the effect of the drugs. But it was also felt that the breath pattern changes suggested that the dose given had some effect and that the dose given was not far from a useful effect. It was therefore agreed, at the next opportunity, to increase the dose to near the top end of the clinical range used in captive marine mammals: 0.1 mg/kg for both midazolam and butorphanol, with a potential supplemental dose of 0.025 mg/kg of both drugs if indicated.

On March 6^th^ 2009 21.8 nm N of Cape Canaveral, FL, USA. the plane relocated the whale at 10∶34. Two darts were implanted delivering 0.1 mg/kg midazolam HCl and 0.1 mg/kg butorphanol (Table 1). Dart 1 was delivered at 11∶33 low on the right flank with full almost perpendicular penetration. The second dart was delivered at 11∶43 about 301cm ventral and cranial to the first dart. The buoy for dart 1 trailed behind the whale's flukes and remained there until about 12∶35 when the cutting boat observed the dart buoy receding from the whale. It was recovered along with the dart. The dart sleeve had been fully compressed in to the rubber stopper showing full penetration, but with the syringe body lying caudally, suggesting a bend in the needle at the skin surface comparable to observations of darts deployed in other cases [13]. Subsequent examination showed about 5 ml of a watery dark red liquid in the dart barrel. Cytology from a smear showed peracute hemorrhage, with an abundance of eosinophilic, globular material consistent with the drugs delivered.

**Figure 3:**
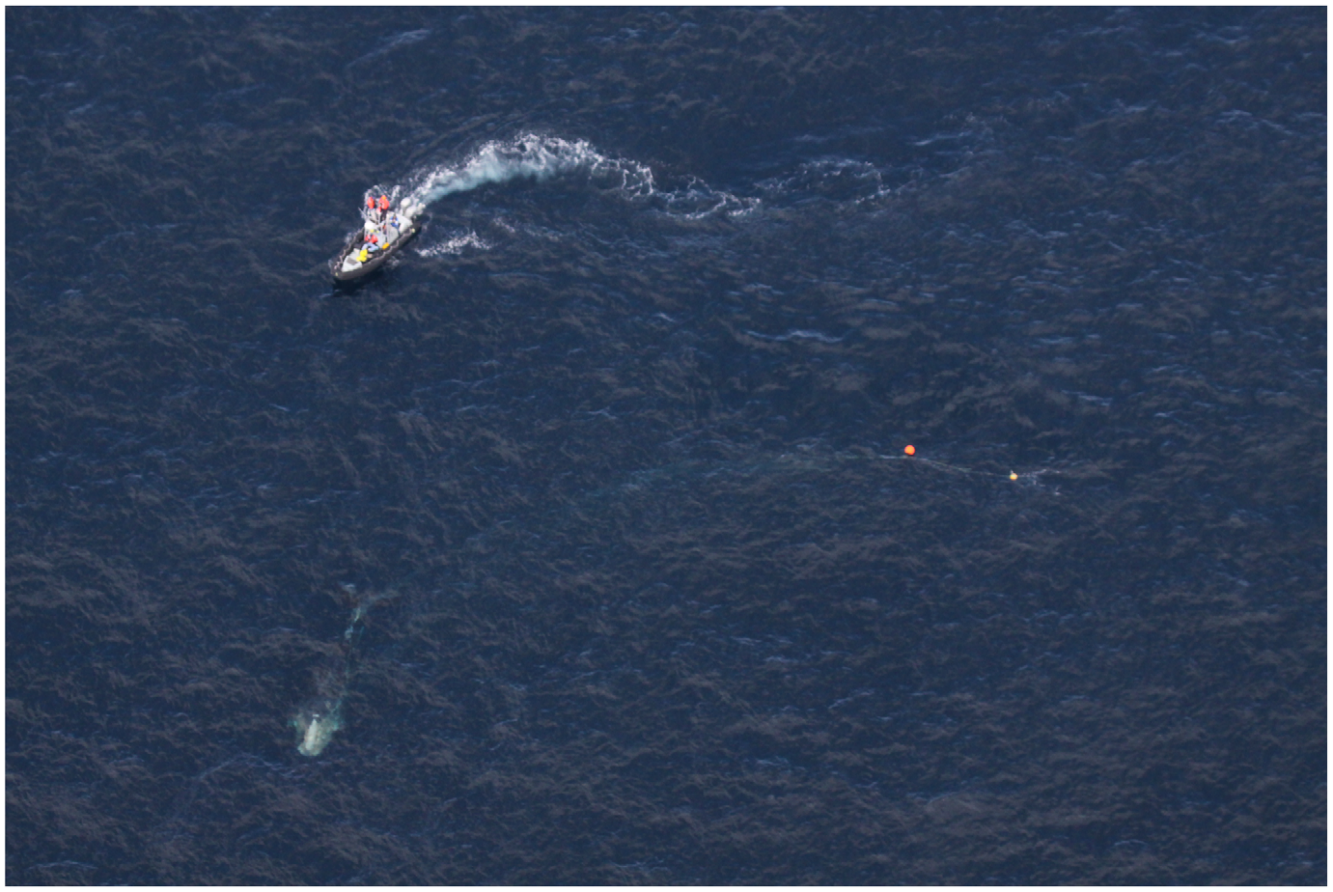
Right whale #3311 swimming at 11:30 on March 6^th^ 2009 prior to sedation. The whale is in the bottom left of the image, and is towing a line with buoys shown to the middle right. Note the sharp turn shown by the line, away from the boat attempting to approach the whale at top left. Photograph credit Georgia Dept Natural Resources/Wildlife Trust.

**Figure 4:**
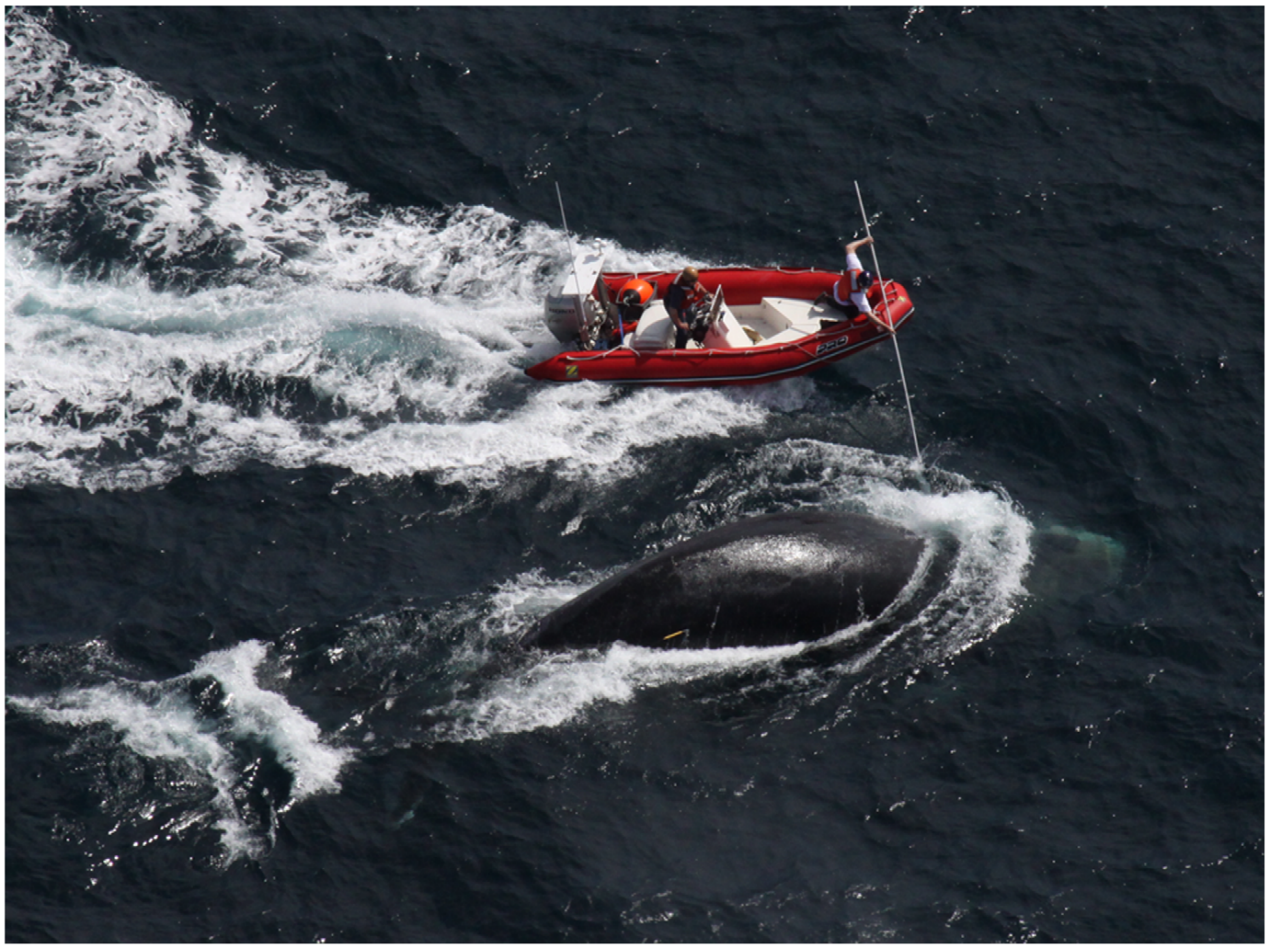
Right whale #3311 swimming at 12:42 on March 6^th^ 2009 after sedation. Whale is shown an hour after sedative administration showing a marked reduction in boat avoidance as compared to the behavior shown in Figure 3. Photograph credit Georgia Dept Natural Resources/Wildlife Trust.

At 12∶06 the plane reported that before injection the whale had been travelling at 1.5 knots and avoiding the sedation vessel (Figure 3), and after injection the speed increased to 2 knots. It travelled 0.63 nm in 18 minutes. The plan to assess the degree of sedation was to make a boat approach and observe the persistence of the characteristic strong boat avoidance reaction that was ubiquitous in previous approaches to the animal. At 12∶30 an approach was made. By 12∶43 three cutting approaches had been made, the first of which did not allow for the spring loaded cutting knife to make contact with the whale. Contact was made on three subsequent approaches. The first and second cuts were a miss but a cut to the head wrap was made at the third cutting attempt (Figure 4). It was the opinion of the cutting boat crew (CS (driver) and JS (cutter) that the behavior of the whale was markedly different.

**Figure 5:**
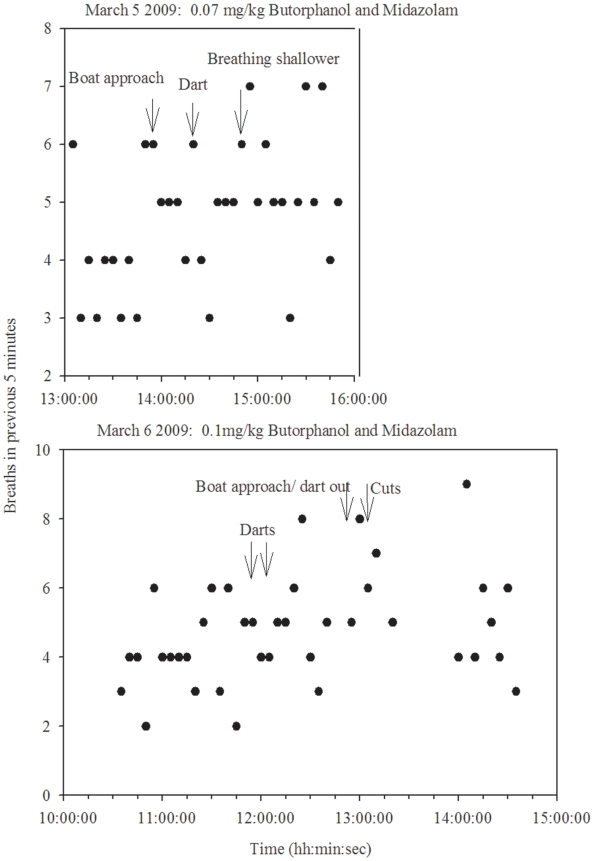
Number of breaths made by right whale #3311 in preceding five minutes, compared before and after sedation.

Breath counts in each five minute period were compared before and after sedative injection on March 5^th^ and 6^th^ 2009 for whale #3311. A Mann-Whitney rank sum test showed a significant increase in respiratory frequency following drug delivery on March 6^th^, but not March 5^th^ (Figure 5 and Table 2). After being freed of the majority of the line and the tracking buoy, the whale began to move away from the disentanglement area. The plane that was responsible for maintaining contact with the whale was running low on fuel, and daylight, and was required to leave the scene. The darting boat made one more parallel pass to take a set of photos of the rope damage and to observe the animal's behavior.

**Table 2:** Number of breaths per 5 minutes over 75 observed minutes before and after first dart delivery to right whale # 3311.

	Mean (± SD) number of breaths per 5 minutes during a 75 minute period	
	March 05 2009	March 06 2009
Before darting	4.3±1.1	4.0±1.3
After darting	5.1±1.2	5.3±1.5
Mann-Whitney Rank Sum Test	0.076	**0.018**

The most notable change in behavior, post-sedation on March 6^th^ 2009, was the whale's apparent inability or desire to move away from the approaching vessel. During previous events, and to a lesser degree on March 5^th^ 2009, the whale was able to cease an intended surfacing 4–5 meters below the surface, lower its head and lift its flukes to make the sharp left turn for avoidance measures. Post-sedation on March 6^th^ 2009, the whale seemed indifferent to interrupting a surfacing for a second breath once it had begun to make this surfacing. This made placement of the boat next to the whale's head and planning for the materials needed for cutting much more achievable. Timing of surfacing was essential for managing these short periods of interaction which were directed with the help of the aircraft crew above. It was noting this repetition of a second breath that enabled the disentanglement team to make cuts. Shallowness of breath was also mentioned by the sedation team as one of the observed effects after darting. The whale remained under observation for 2 hours. Once freed from the line and telemetry buoy the whale's swimming speed increased and respirations became deeper. It was elected not to reverse the butorphanol at this point.

## Discussion

Anesthesia, the act of reducing consciousness to a point where surgery can be undertaken, is rarely attempted in cetaceans as it is fraught with risk, given our very limited knowledge of cetacean pharmacokinetics and the complexity of managing their breath hold dive reflex, bradycardia, and high tolerance to acidosis [5]. In contrast, sedation has been used more widely in captive and beached cetaceans to facilitate procedures, since it reduces the subject's resistance without the risks associated with loss of consciousness. Sedating large whales at sea in our study required enhanced tractability, without any loss of ability to swim and respire. The major risk of excessive sedation while still swimming and diving was uncontrolled inhalation of water. Since there had been no previous attempt to intervene medically with free ranging cetaceans at sea, we had to develop drug delivery and use protocols that achieved a sedated free swimming cetacean with reduced resistance to being handled, while still swimming and respiring voluntarily with no loss of equilibrium or inhalation of water.

The initial choice of a cantilevered pole syringe was based on the assumption that an adequately large syringe and needle length could not be designed and built to be sufficiently accurate as a ballistic device. The pole syringe proved to be functional, but the risk of being mechanically attached to the whale if the syringe did not release from the pole, and the logistic complications of deploying a cantilever system offshore, lead to a desire to revisit the drug delivery system design and subsequent development of the ballistic system built by Paxarms. One of the issues in the early trials of this system was the inertia of the loaded syringe barrel bending the implanted needle. Addition of a carbon fiber needle liner solved this problem.

Concern about loaded darts that missed their target and retained concentrated drug to be encountered by a third party, lead to development of a tethered dart.

The choice of drugs was central to project development. Marine mammal clinicians, with experience in sedating smaller cetaceans, and veterinary anesthesiologists weighed cost against likely efficacy. First priority was to utilize drugs that would result in the most likely chance of success during disentanglement. At the outset butorphanol was regarded as prohibitively costly. However, given that it facilitated disentanglement in under two hours, its costs may be offset by reductions in the number of days at sea required for successful disentanglement.

The actual dose will always depend on an approximate estimate of mass in the field, even though an intended mg/kg of drug is prescribed. Most right whale cases will involve catalogued individuals, so their age will often be known. Thus available mass, length and age relationships from stranded animals [2] are the best current option for mass estimation.

The reduction in boat aversion in whale #3311 on March 6^th^ 2009 may have occurred as early as 30 minutes after the second dart was injected, but it took an hour to close the distance to the whale and prepare the cutting tools for cutting approaches. Care must be taken to remain within striking distance of a sedated whale while remaining far enough away to ensure that boat noise is not “pushing” the whale, thus reducing the effects of sedation. This was especially true in the case of whale #3311. The closing time for the 5.5 m RHIB was much increased due to deteriorating sea conditions. In summary the whale continued to breathe and dive, but was no longer avoiding boat approaches as effectively as it was prior to sedation.

Comparing the behavior of whale #3311 during approaches made on the third disentanglement event, spanning March 5^th^ and 6^th^ 2009, with behavior observed on January 22^nd^ and 23^rd^ 2009 and on February 1^st^ 2009, it should be noted that the reduced physical condition of the whale may have contributed to slower movement in the water during close approaches and decreased the time the whale could remain submerged under “close approach stress” after a single breath surfacing. Sedation may also reduce the depth of inhalation and deep diving avoidance behavior resulting in more time near the surface and higher respiratory rates. Increased respiration rates have been seen in other cetaceans which spend more time near the surface. However prior to the final sedation attempt this only marginally reduced the whale's ability to avoid the very close approaches needed for cutting. The initial desire to stop the animal for extended work was not realistic based on behavioral responses from captive individuals given similar dosages. In this regard, a majority of sedated captive dolphins, beluga, *Pseudorca* or *Orca* have maintained their capability for movement and respirations. Variation in response may reflect individual response to the drugs, drug dosage and the size of the sedation pools used for administration and observation during onset period of the sedatives. However there have been observations of a decrease in resistance to handling and manipulation, and subtle changes in respiratory quality and rate.

Reversal of midazolam with flumazenil was not practical since this agent is not available in a concentrated form and would have taken 400 mls minimum, equal to 7 additional darts. Flumazenil also has a short half life, making the risk of re-induction real. The evaluation of a whale’s need for reversal with these sedatives is largely based on the behavioral response, though there may be concern for the change in drug effects due to reduced stress levels following disentanglement. Some animals may be overly sedated after the completion of a procedure. It may be prudent, when possible, to partially reverse the combined sedative effect by use of naltrexone at the completion of the procedure since follow up is often difficult without satellite tracking. Another option is to decrease the midazolam dose to 0.075 mg/kg while maintaining the butorphanol dose at 0.1 mg/kg until more individuals have been studied. As of February 19^th^ 2010 whale #3311 has not been re-sighted dead or alive. Thus the success of the disentanglement remains unknown. While use of the sedative system did enhance disentanglement effectiveness in this case the enhancement to survival remains unknown at this time. This was a conservative sedative approach. There are more powerful sedative agents available that could have potentially slowed the animal but these have not been successfully used in other cetaceans and would be more likely to lead to a negative animal outcome. The choice of two reversible drugs used successfully in other cetacean species minimized the risk to the animal, but the true impact of this new technique cannot be assessed until it has been applied to a number of other large whales.
